# Crowding-out effect of out-of-pocket expenditure on non-communicable diseases in sub-Saharan Africa: a Nigerian case study

**DOI:** 10.1093/inthealth/ihaf117

**Published:** 2025-11-20

**Authors:** Adelakun Odunyemi, Hamid Sohrabi, Khurshid Alam

**Affiliations:** Health Administration, Policy and Leadership, Murdoch Business School, Murdoch University, Perth, 6150, Western Australia; Hospitals Management Board, Akure, Ondo State, Nigeria; Centre for Healthy Ageing, Murdoch University, Perth, 6150, Western Australia; Health Administration, Policy and Leadership, Murdoch Business School, Murdoch University, Perth, 6150, Western Australia

**Keywords:** crowding-out effect, food security, household consumption, Nigeria, non-communicable disease, out-of-pocket health expenditure

## Abstract

**Background:**

This study investigates the crowding-out effect of out-of-pocket (OOP) health expenditures for non-communicable diseases (NCDs) on household consumption in Nigeria, a critical issue in sub-Saharan Africa (SSA) given the escalating prevalence of NCDs and associated high care costs.

**Methods:**

Using data from the 2018/2019 Nigerian Living Standard Survey, we employ a Generalised Method of Moments estimator and conditional Engel curve equations derived from the Quadratic Almost Ideal Demand System to analyse the impact of NCD and non-NCD OOP expenditures on 13 household consumption categories.

**Results:**

Despite representing <2% of household budgets, NCD OOP expenditures significantly crowded out essential spending, particularly on healthier food options (fruits, vegetables, protein) and cooking energy sources, disproportionately affecting lower-income households. Intriguingly, discretionary spending on sugar, alcoholic/sugary beverages, entertainment (including tobacco) and meals outside the home remained unaffected, possibly indicating reliance on these fast meal alternatives due to limited cooking fuel access. Middle-income households also experienced crowding out of staples and education expenditures.

**Conclusions:**

These findings highlight the urgent need for universal health coverage to reduce OOP burdens, alongside targeted interventions addressing NCD burden, dietary quality, the energy crisis and health inequities in Nigeria and, by extension, SSA.

## Introduction

The global increase in out-of-pocket (OOP) health expenditure threatens the achievement of the health-related Sustainable Development Goals (SDGs) by 2030. According to the WHO, the proportion of the population spending >10% of their household budget as OOP health expenditure has worsened since the beginning of the SDGs, reaching 13.5% (about 1 billion people) in 2019 from 12.7% in 2015. OOP health expenditure is a precursor for poverty, pushing about 4.4% of the world’s population into extreme poverty in 2019.^1^

OOP health expenditure places a significant financial burden on household welfare in sub-Saharan Africa (SSA), where OOP as a proportion of total health expenditure is approximately 36% (>15–20% global cut-off point).^1^ Some SSA countries, including Nigeria’s record in 2019, exceeded 70%.^2^ Nigeria has one of SSA’s highest OOP health expenditures.^3^ Nigeria’s OOP health expenditure rose from 60.2% in 2000 to 70.5% in 2019. By 2021, it had increased to 76.2%, compared with the 33% African average and a much lower 28.2% world average.^4^ Widespread inequality in health financing systems, lack of prepayment mechanisms and lack of social protection are responsible for Nigeria’s high OOP health expenditure.^3^ Meanwhile, government health spending as a percentage of gross domestic product has remained abysmally low, between 0.51% and 0.59% since 2015 and >90% of private health expenditure is spent as OOP.^4^

Because of OOP spending, a household may need to sacrifice essential household consumption expenditures to access healthcare. The extent to which OOP health expenditure displaces household non-medical consumption is called the crowding-out effect.^5,6^ With progressively high OOP health expenditures, as seen with non-communicable diseases (NCDs), poor households are also compelled to reduce these essential consumptions.^7^ Nigeria dominates the picture of the high financial burden of NCDs in SSA, driving catastrophic health expenditure and impoverishment.^8,9^ This leads to a household’s living standard and food security deterioration, trapping them in poverty.^9^

Because the cornerstone of all measures of financial protection is the ability of households to protect non-medical consumption from OOP health expenditure,^8^ crowding-out effects of OOP health expenditure should be included in the measures of financial risk protection. Unfortunately, studies on the crowding-out effect of OOP health expenditure, especially for NCDs, are lacking in SSA.^8^ Only a handful of studies on the topic exist, and most are not NCD-specific.^5,6,10,11^ To the best of our knowledge, only two studies have addressed the NCD-related crowding-out effect. By comparison, one focused on the cost of NCD medications,^12^ and the other considered NCD households (with non-NCD spending) without disaggregating NCD-specific expenditure.^7^

This study evaluates the crowding-out effect of OOP spending on NCDs on household consumption. Despite the unacceptably high food insecurity, poverty and high OOP health spending in SSA, no studies have assessed the impact of health spending on household consumption.^8^ To the best of our knowledge, this study is the first empirical evidence of the impact of NCD OOP health expenditure on household consumption in SSA. OOP health expenditure constitutes a substantial proportion of total health spending in many SSA countries, including Nigeria, where it reached 76.2% by 2021.^13^ There is limited empirical evidence quantifying how NCD-related OOP expenditures crowd out household consumption in Nigeria and SSA.^8^ Nigeria was selected due to its large population, high OOP health spending, low government health investment (3–5% of the budget, far below the Abuja Declaration benchmark of 15%) and rising NCD prevalence, which exacerbates the risk of poverty through catastrophic health expenditure.^8,14,15^ Therefore, understanding these crowding-out effects is crucial for informing policy responses to health inequities and poverty traps associated with NCDs in the SSA region.

Unlike previous studies, we disaggregated spending on NCDs and non-NCDs, estimating their crowding-out effects separately. This helps to compare what each expenditure crowds out (or in). Moreover, compared with similar studies, we employed a more consistent and efficient Generalised Method of Moments (GMM) estimator to account for heteroscedasticity common in consumption data. Also, due to endogeneity, the method used in some studies^11^ could result in biased and inconsistent estimates.

## Methods

### Data description

#### Household consumption expenditure

The data used in this study were obtained from the 2018/2019 Nigerian Living Standard Survey (NLSS) conducted by Nigeria’s National Bureau of Statistics (NBS). The nationally representative survey sampled 22 200 households using a stratified multistage random sampling method. NLSS is currently the most recent comprehensive nationally representative publicly available dataset for household consumption and health expenditures in Nigeria. Household data collected include sociodemographics, education, health access, labour, housing, assets and consumption. Household consumption items were divided into 13 mutually exclusive categories. We classified food items into six categories: staple foods (grains, rice, maize, baked and starchy foods), proteins (meat, fish, poultry, dairy and pulses), fruits and vegetables, sugar and beverages (alcoholic and non-alcoholic drinks), oil and other foods and meals consumed outside the home. Non-food items were broadly grouped into seven categories: housing (actual rent), clothing (including footwear), education, energy (electricity and fuel), transportation, durable goods, as well as personal services and entertainment (including newspapers, magazines, cinemas, leisure and tobacco). These categories follow the standard classification used in household consumption studies.^5,10,12^ The expenditure (non-health) on these 13 items is calculated as the annual value in Nigerian Naira (₦) and converted to international dollars using the 2019 average official exchange rate of ₦306.92 to US$1.^16^

The health module allowed us to extract total OOP health expenditures, subdividing them into NCD and non-NCD spending based on disease categorisation, which followed previously validated methods.^9^

### Analytical framework for crowding-out effects

We conducted preliminary (unadjusted) t tests on the difference in mean budget shares of expenditure between spender and non-spender households for each of the 13 consumption items. We analysed this for all diseases, NCDs and non-NCDs. A statistically significant value (t-value>2) represents the coefficient of the crowding-out (negative sign) or crowding-in (positive sign) effect of OOP health expenditures on the consumption item.

Then we estimated the crowding-out (or crowding-in) effect of OOP health expenditures on the consumption items using the consumer theory developed by Pollak in 1969,^17^ adjusting for household characteristics:


(1) \begin{equation*}
{\rm Max}U = U ( {{q}_1, \ldots {{\bar{q}}}_n,Y;a} )\ s.a.\ \mathop \sum \nolimits_{n - 1}^{i = 1} {p}_i{q}_i = M\& {q}_n = {\bar{q}}_n,
\end{equation*}

where ${q}_i$ and ${p}_i$ are the quantity and price of consumption item *i*, ${\bar{q}}_n$ denotes the demand for healthcare, *Y* is the total household expenditure and $M = Y - {p}_n*{\bar{q}}_n$ (where ${p}_n$ is the cost of healthcare).

Therefore, solving for *n* – 1, conditional on the healthcare consumption, produces the following demand function:


(2) \begin{equation*}
{q}_i = {g}^i\ \left( {{p}_1, \ldots ,{p}_{n - 1},\ M;\ {{\bar{q}}}_n;h} \right)\ \forall \ i\ \not= \ n.
\end{equation*}

This implies that the demand function for any household item ${q}_i$ is conditional on the price of all other household items (except the conditioning item, healthcare ${q}_n$), total non-health expenditure *M* after OOP health expenditure is removed.^18^

## Specification of the econometric model

The form commonly used to estimate the above equation is the conditional Engel curves from the Quadratic Almost Ideal Demand Systems (QUAIDS) developed by Banks et al. in 1997.^19^ It has been used in crowd-out literature on health,^5–7,10,12^ sugar^20^ and tobacco and alcohol expenditures.^21^ While alternative techniques, such as genetic matching, propensity score matching and fractional logistic regression, have also been employed,^22,23^ the QUAIDS model is preferred for several reasons. QUAIDS directly models household budget allocation dynamics, effectively addresses potential endogeneity issues, accounts for non-linear relationships between variables and accommodates the proportional nature of household budget shares.^21^ Furthermore, matching methods are generally less effective for analysing continuous, interdependent spending behaviours and lack the structural flexibility needed to simulate the impacts of policy interventions.^5,18^

The QUAIDS follows consumer economic theory, which includes a quadratic expenditure term.^19,21^ For a consumption item *i* of household *h*, the Engel curve econometric specification takes the basic form:


(3) \begin{equation*}
{w}_{ih} = {\beta }_{0i} + {\beta }_{1i}OOP + \ {\beta }_{2i}\left( {lnM} \right) + {\beta }_{3i}{\left( {lnM} \right)}^2 + {\beta }_{4i}{X}_h + {\varepsilon }_{ih},
\end{equation*}

where ${w}_{ih}$ stands for non-health budget shares of each consumption item *i*, using *M*, which is the total non-health expenditure. $OOP$ is the total OOP health expenditure, OOP health expenditure on NCDs or non-NCDs (estimated separately); ${X}_h$ is the vector of household socioeconomic and demographic characteristics identified from the literature.^6,7,11,12,22^ This vector includes characteristics of the household head, such as age (log-transformed), sex, marital status (single, married, divorced, widowed) and highest level of education attained (none, primary, secondary, tertiary). Additional household characteristics included are the number of equivalent adults (adjusting for household size and economies of scale in consumption), covariates indicating household dependencies (working adult ratio, dependency ratio [proportion of children and people aged >65 y], proportion of women of reproductive age [15–49 y] and mean disability index [calculated as the unweighted mean of the sum of six binary questions relating to difficulty with seeing, hearing, walking or climbing, remembering or concentrating, self-care and communicating]) and variables indicating the socioeconomic status of the household, such as participation in any safety nets or social programmes (assistance), acquisition of remittances and loans, types of toilets and cooking stoves used and place of residence (rural/urban locations and geopolitical zones, accounting for regional differences in prices and consumption preferences). $lnM$ and $ln{M}^2$ represent the natural logarithms of M and M squared, respectively, and ${\varepsilon }_{ih}$is the idiosyncratic error term. To isolate the effect of non-NCD expenditure, we included it as a consumption category when estimating the crowding-out effect of NCDs and vice versa. We transformed age, travelling and waiting time, M and M2, into their natural logs to account for non-linear effects. The coefficient of interest is ${\beta }_{1i}$. A negative (positive) value of ${\beta }_{1i}$ indicates OOP health expenditure crowds out (crowds in) the share of expenditures devoted to item *i*.

The value of ${\beta }_{1i}$ is usually small because it refers to a percentage point (pp) change in the annual budget share of expenditure on an item relative to 1 increase in OOP health expenditure.

Because household expenditure decisions are made contemporaneously, OOP health and total non-health expenditures are likely endogenous due to simultaneity and multicollinearity. Therefore, the above equation cannot be estimated using the ordinary least squares method. An instrumental variable (IV) method is required. A suitable IV must be exogenous, correlating with the endogenous regressors but uncorrelated with the error term or dependent variable (i.e. other consumption items).^21^

As in previous studies,^5,7,10,12^ we used a natural log of total household expenditure (including OOP health expenditure) and its square to instrument for total non-health expenditure (*M*). We instrumented OOP health expenditures using a natural log of travel and waiting times. Travelling and waiting times to healthcare facilities have been shown to impact healthcare access, costs and outcomes, including hospitalisation rates,^24^ and they are not directly influenced by factors affecting OOP health expenditure. We confirmed the validity and relevance of our IVs for all equations using the Cragg–Donald Wald F-statistic for first-stage regression, Shea’s partial R2 diagnostic test, Kleibergen–Paap rank LM statistic and Sanderson–Windmeijer multivariate F test (Supplementary Table 1). The absence of significant heterogeneous preferences between spender and non-spender, suggested by Vermeulen’s (2012) consumer separability concept,^25^ was confirmed by Wald tests.

Although the Durbin–Wu–Hausman test result is mixed, OOP and non-health expenditures are usually endogenous.^11^

The choice of the analytical method for Equation (3) depends on the error terms’ heteroscedasticity.^21^ Typically, equation-by-equation instrumental variables estimation, which is a Two-Stage Least Square (2SLS) plus IV, also called a ‘system 2SLS estimator’, or a system of equations instrumental variable estimation (similar to seemingly unrelated regression plus IV), which is effectively a Three-Stage Least Square (3SLS),^6,7,10,12^ is used. However, these methods are only efficient if error terms are homoscedastic^5,6,10^; otherwise, the GMM estimator is preferred.^21^ Therefore, many recent crowding-out studies employed this method.^26,27^ Pagan–Hall statistic and Breusch–Pagan Lagrange Multiplier tests on our model reveal heteroscedasticity. Therefore, we adopted a GMM estimator method. To reduce computation time, we employed *ivreg2* with the gmm2s option in Stata to perform two-step efficient GMM estimation, which uses an optimal weighting matrix to account for heteroscedasticity or clustering. We also applied heteroscedasticity-consistent robust standard errors clustered at primary sampling unit.

NCD OOP expenditure crowding-out was also compared across household socioeconomic tertiles/strata (SES).

All statistical analyses were performed using Stata/MP 17 for Windows (StataCorp, TX, USA).

## Results

### Distribution of household characteristics and expenditures by socioeconomic status

Table 1 presents household characteristics and expenditures by SES. The mean age of household heads was similar across SES. Female-headed households constituted <25% across all SES, with >70% married. Literacy beyond primary school exceeded 50% overall, with the lowest literacy rate of 26.43% (95% CI 24.35 to 28.52%) in the lowest SES group. The North-West and South-West geopolitical zones had the highest concentrations of poor (34.81%; 95% CI 32.48 to 37.14%) and rich (43.26%; 95% CI 39.18 to 47.33%) households, respectively. Rural residence was more prevalent among the lowest SES (82.04%; 95% CI 81.02 to 83.06%) compared with the highest SES (39.30%; 95% CI 37.83 to 40.77%). Improved sanitation access was significantly higher in the highest SES (70.09%; 95% CI 66.76 to 73.43%) than in the lowest SES (36.91%; 95% CI 35.15 to 38.67%).

**Table 1.** Distribution of household characteristics and expenditures by socioeconomic status

		Socioeconomic status^a^		
Characteristics/expenditures	All mean/proportion (95% CI)	Lower mean/proportion (95% CI)	Middle mean/proportion (95% CI)	Upper mean/proportion (95% CI)
**Household head’s characteristics**				
Age, y (mean)	49.95 (49.46–50.45)	48.61 (47.95–49.28)	49.93 (49.09–50.78)	52.13 (50.95–53.31)
Female head (proportion)	13.63% (12.42–14.83%)	9.34% (7.78–10.90%	13.89% (11.90–15.88%)	20.11% (17.09–23.14%)
Marital status (proportion)				
Married	84.87% (83.60–86.13%)	89.02% (87.36–90.67%)	84.90% (82.80–86.99%)	78.18% (75.02–81.33%)
Divorced	2.73% (2.12–3.34%)	2.00% (1.11–2.89%)	3.22% (2.14–4.29%)	3.21% (1.92–4.50%)
Widowed	10.42% (9.34–11.49%)	7.74% (6.39–9.10%)	9.70% (8.02–11.39%)	15.73% (12.90–18.56%)
Single	1.98% (1.50–2.46%)	1.24% (0.69–1.79%)	2.18% (1.26–3.10%)	2.88% (1.78–3.99%)
Highest level of education (proportion)				
None	14.16% (13.09–15.23%)	26.43% (24.35–28.52%)	8.43% (6.99–9.88%)	2.78% (1.59–3.97%)
Primary	33.95% (32.35–35.56%)	37.27% (34.91–39.63%)	36.32% (33.53–39.10%)	25.23% (21.97–28.49%)
Secondary	33.03% (31.40–34.67%)	27.43% (25.22–29.64%)	38.14% (35.33–40.95%)	34.63% (30.85–38.41%)
Tertiary	18.85% (17.44–20.27%)	8.86% (7.53–10.20%)	17.11% (14.94–19.29%)	37.36% (33.49–41.23%)
**Household characteristics**				
Geopolitical zone (proportion)				
North-Central	13.77% (12.74–14.80%)	12.22% (10.77–13.68%)	16.71% (14.82–18.60%)	12.01% (9.90–14.12%)
North-East	11.56% (10.71–12.40%)	18.74% (17.10–20.38%)	9.28% (7.99–10.56%)	3.35% (2.43–4.27%)
North-West	21.24% (19.90–22.59%)	34.81% (32.48–37.14%)	16.07% (13.93–18.21%)	7.00% (5.20–8.80%)
South-East	16.46% (15.28–17.63%	18.34% (16.47–20.21%)	18.43% (16.37–20.50%)	10.58% (8.52–12.63%)
South-South	16.35% (15.09–17.61%)	10.22% (8.60–11.83%)	17.99% (15.84–20.13%)	23.81% (20.70–26.92%)
South-West	20.62% (18.91–22.34%)	5.67% (4.12–7.22%)	21.52% (18.67–24.37%)	43.26% (39.18–47.33%)
Rural location (proportion)	60.31% (59.47–61.15%)	82.04% (81.02–83.06%)	59.60% (58.19–61.01%)	39.30% (37.83–40.77%)
Adult equivalent	4.90 (4.83–4.98)	5.68 (5.55–5.80)	4.72 (4.60–4.84)	3.94 (3.79–4.08)
Work ratio	0.73 (0.72–0.74)	0.73 (0.72–0.75)	0.75 (0.73–0.76)	0.69 (0.66–0.71)
Adult sex ratio (male: female)	1.05 (1.02–1.07)	1.02 (0.99–1.06)	1.08 (1.04–1.12)	1.04 (0.99–1.10)
Dependent ratio	0.45 (0.44–0.46)	0.52 (0.51–0.53)	0.44 (0.43–0.45)	0.35 (0.33–0.37)
Average disability (mean)	1.51 (1.48–1.54)	1.53 (1.49–1.57)	1.51 (1.46–1.56)	1.48 (1.41–1.54)
women of reproductive age (proportion)	50.18% (49.25–51.11%)	47.23% (46.08–48.38%)	49.73% (48.22–51.25%)	55.56% (53.18–57.95%)
Received remittance (proportion)	58.77% (57.10–60.44%)	48.46% (46.03–50.89%)	62.30% (59.53–65.07%)	70.17% (66.61–73.72%)
Received assistance (proportion)	15.55% (14.38–16.72%)	20.49% (18.56–22.41%)	15.72% (13.73–17.70%)	7.39% (5.34–9.45%)
Received loan (proportion)	29.49% (27.97–31.02%)	27.99% (25.87–30.11%)	32.69% (30.02–35.35%)	27.29% (23.91–30.68%)
Source of sanitation (proportion)				
None	21.54% (20.26–22.81%)	28.04% (25.96–30.13%)	21.84% (19.64–24.04%)	10.68% (8.64–12.72%)
Unimproved	41.56% (39.90–43.21%)	57.62% (55.21–60.02%)	39.22% (36.47–41.97%)	19.23% (16.37–22.08%)
Improved	36.91% (35.15–38.67%)	14.34% (12.43–16.25%)	38.94% (36.02–41.87%)	70.09% (66.76–73.43%)
Type of cooking stove (proportion)				
Polluted source	85.73% (84.31–87.15%)	97.95% (97.03–98.88%)	89.26% (87.13–91.40%)	61.07% (57.14–65.01%)
Clean source	14.27% (12.85–15.69%)	2.05% (1.12–2.97%)	10.74% (8.60–12.87%)	38.93% (34.99–42.86%)
**Household consumption expenditure**				
Total OOP health (US$)	195.12 (185.82–204.42)	143.00 (138.27–147.73)	199.52 (190.74–208.30)	242.85 (216.85–268.85)
Budget share of total OOP health expenditure	6.24% (6.10–6.38%)	6.96% (6.77–7.16%)	6.45% (6.21–6.69%)	5.30% (5.02–5.58%)
Total non-NCD OOP health expenditure (US$)	133.51 (125.43–141.59)	104.20 (100.17–108.23)	137.16 (130.03–144.30)	159.17 (136.37–188.97)
Budget share of non-NCD OOP health expenditure	4.32% (4.21–4.44%)	5.10% (4.93–5.26%)	4.46% (4.26–4.65%)	3.42% (3.21–3.63%)
Total NCD OOP health expenditure (US$)	42.07 (38.10–46.05)	23.39 (21.45–25.32)	40.04 (36.10–43.98)	62.79 (51.73–73.85)
Budget share of NCD OOP health expenditure	1.29% (1.21–1.37%)	1.17% (1.07–1.27%)	1.30% (1.18–1.42%)	1.40% (1.22–1.59%)
Food (at home)^b^				
Total staples (US$)	591.55 (584.33–598.77)	579.42 (569.58–589.26)	635.70 (623.85–647.54)	559.54 (544.39–574.71)
Budget share of staples	21.71% (21.52–21.90%)	29.22% (28.93–29.52%)	21.56% (21.30–21.82%)	14.34% (14.09–14.59%)
Total protein (US$)	404.18 (397.31–411.06)	256.62 (250.96–262.26)	428.42 (419.02–437.82)	527.54 (510.73–544.34)
Budget share of protein	13.27% (13.14–13.40%)	12.45% (12.26–12.65%)	14.15% (13.94–14.37%)	13.21% (12.95–13.47%)
Total fruits and vegetables (US$)	220.63 (217.87–223.40)	176.40 (172.93–179.87)	233.76 (229.37–238.14)	251.75 (245.75–257.74)
Budget share of fruits and vegetables	8.03% (7.95–8.10%)	9.12% (8.99–9.25%)	8.20% (8.08–8.31%)	6.76% (6.64–6.88%)
Total sugar and beverage (US$)	72.65 (70.89–74.40)	39.63 (38.19–41.08)	68.61 (66.24–70.99)	109.70 (105.53–113.86)
Budget share of sugar and beverages	2.26% (2.22–2.30%)	1.88% (1.83–1.94%)	2.20% (2.13–2.26%)	2.70% (2.62–2.79%)
Total oil and others (US$)	158.81 (156.77–160.85)	136.85 (134.56–139.15)	168.14 (164.83–171.44)	171.44 (166.85–176.02)
Budget share of oil and others	5.96% (5.91–6.02%)	7.35% (7.26–7.44%)	5.95% (5.86–6.03%)	4.60% (4.51–4.69%)
Total meals (outside home) (US$)	365.32 (355.74–374.90)	181.96 (175.91–181.00)	330.56 (319.70–341.41)	583.47 (559.35–607.59)
Budget share of meals (outside home)	11.91% (11.69–12.12%)	8.56% (8.31–8.81%)	11.20% (10.87–11.52%)	15.96% (15.48–16.44%)
Total housing (actual rent) (US$)	57.46 (53.78–61.15)	10.67 (9.47–11.88)	42.00 (38.81–45.19)	119.72 (109.67–129.77)
Budget share of housing (actual rent)	1.53% (1.46–1.61%)	0.54% (0.48–0.59%)	1.38% (1.28–1.49%)	2.68% (2.50–2.86%)
Total clothing and footwear (US$)	143.99 (141.30–146.68)	97.85 (95.77–99.94)	136.95 (133.53–140.37)	197.17 (190.62–203.72)
Budget share of clothing and footwear	4.76% (4.71–4.81%)	5.04% (4.96–5.12%)	4.51% (4.42–4.59%)	4.74% (4.64–4.84%)
Total education expenditure (US$)	190.39 (183.03–197.74)	84.96 (83.39–88.53)	186.00 (178.23–193.77)	300.21 (280.38–320.04)
Budget share of education	4.55% (4.44–4.67%)	3.73% (3.59–3.86%)	5.00% (4.82–5.18%)	4.94% (4.68–5.19%)
Total energy (US$)	160.24 (155.90–164.58)	71.02 (68.63–73.42)	146.78 (142.25–151.30)	262.94 (251.68–274.20)
Budget share of energy	4.74% (4.66–4.81%)	3.38% (3.28–3.48%)	4.81% (4.69–4.93%)	6.02% (5.87–6.18%)
Total transportation (US$)	161.74 (156.88–166.60)	68.28 (64.76–71.81)	146.44 (140.48–152.41)	270.51 (258.87–28 214)
Budget share of transportation	4.98% (4.86–5.09%)	3.19% (3.05–3.32%)	4.85% (4.67–5.03%)	6.90% (6.65–7.14%)
Total durable items (US$)	124.41 (121.75–127.07)	84.16 (82.49–85.82)	118.70 (116.22–121.18)	170.38 (163.21–177.54)
Budget share of durable items	4.26% (4.21–4.30%)	4.44% (4.37–4.51%)	4.10% (4.04–4.17%)	4.23% (4.14–4.32%)
Total personal services and entertainment (US$)	210.34 (196.07–244.61)	84.56 (82.40–86.73)	175.14 (170.49–179.79)	371.34 (329.31–413.36)
Budget share of personal services and entertainment	5.80% (5.72–5.89%)	4.13% (4.05–4.22%)	5.65% (5.54–5.76%)	7.63% (7.43–7.82%)
	***n*=22 110**	***n*=9010**	***n*=7208**	***n*=5892**

NCD: non-communicable disease; OOP: out-of-pocket.

Estimates are weighted and based on the Nigeria Living Standard Survey 2018/2019.

95% CIs are shown in parentheses.

a Tertiles of adjusted household expenditure per capita.

b Staples represent rice, maize, grain, baked and starchy foods; protein represents meat, fish, poultry, dairy and pulses; beverages represent alcoholic and non-alcoholic drinks; and entertainment includes newspapers, magazines, cinemas, leisure and tobacco.

While higher SES households spent more on most consumption items, budget share allocation varied. Food, particularly staples (21.71%; 95% CI 21.52 to 21.90%) and protein (13.27%; 95% CI 13.14 to 13.40%), represented the largest budget share for all households, while housing (rent) had the smallest. Higher SES households allocated a smaller budget share to food (except sugar and beverages), but a larger share to non-food items (except OOP health expenditure). Total OOP health expenditure comprised 6.24% (95% CI 6.10 to 6.38%) of total household expenditure, the largest non-food category, with NCD spending representing approximately 21%. The highest SES had a slightly higher NCD OOP expenditure budget share.

### Unadjusted differences in the expenditure share of household items

Supplementary Table 2 shows unadjusted mean budget share differences. Food expenditure, particularly staples, appeared protected (except fruits/vegetables and sugar/beverages). Oil and other food categories seemed protected by OOP health expenditure. NCD spending appeared to protect education and durable items while impacting some food items.

### Adjusted crowding-out effect of out-of-pocket health expenditure

Table 2 presents adjusted crowding-out effects. A negative (positive) coefficient indicates crowding out (crowding in). Total OOP health expenditure crowded out clothing/footwear and crowded in durable items. Essential items (food, meals outside the home, housing, education, electricity and cooking fuel) and some discretionary items (personal services, entertainment [including tobacco] and transportation) were protected.

**Table 2.** Adjusted crowding-out effect of OOP health expenditure for all diseases, non-NCDs and NCDs in Nigeria, 2018/2019

Item name^a^	Coefficient of total OOP health expenditure (95% CI)	Coefficient of non-NCDs OOP health expenditure (95% CI)	Coefficient of NCDs OOP health expenditure (95% CI)
Food (at home)			
Staples	2.29×10^−08^ (−4.85×10^−08^–9.42×10^−08^)	−6.88×10^−08^ (−1.88×10^−07^–5.03×10^−08^)	−3.22×10^−07^*** (−5.47×10^−07^−9.71×10^−08^)
Protein	1.90×10^−08^ (−4.56×10^−08^–8.36×10^−08^)	−5.99×10^−08^ (−1.70×10^−07^–5.03×10^−08^)	−2.97×10^−07^*** (−5.04×10^−07^−8.90×10^−08^)
Fruits and vegetable	−1.71×10^−08^ (−4.96×10^−08^–1.55×10^−08^)	−6.72×10^−08^** (−1.24×10^−07^−1.05×10^−08^)	−2.00×10^−07^*** (−3.08×10^−07^−9.08×10^−08^)
Sugar and beverages	−7.69×10^−09^ (−2.60×10^−08^–1.06×10^−08^)	−2.79×10^−08^* (−5.92×10^−08^–3.28×10^−09^)	−7.73×10^−08^*** (−1.35×10^−07^−1.97×10^−08^)
Oil and others	−1.91×10^−08^* (−4.13×10^−08^–3.01×10^−09^)	−5.35×10^−08^*** (−9.22×10^−08^−1.49×10^−08^)	−1.34×10^−07^*** (−2.06×10^−07^−6.08×10^−08^)
Meals (outside home)	−2.24×10^−08^ (−9.12×10^−08^–4.64×10^−08^)	−1.01×10^−07^* (−2.17×10^−07^–1.39×10^−08^)	−3.17×10^−07^*** (−5.38×10^−07^−9.65×10^−08^)
Housing	9.74×10^−09^ (−1.33×10^−08^–3.28×10^−08^)	1.47×10^−08^ (−2.20×10^−08^–5.14×10^−08^)	1.92×10^−09^ (−5.02×10^−08^–5.41×10^−08^)
Clothing and footwear	−2.93×10^−08^** (−5.17×10^−08^−6.80×10^−09^)	−6.71×10^−08^*** (−1.07×10^−07^−2.71×10^−08^)	−1.57×10^−07^*** (−2.33×10^−07^−8.12×10^−08^)
Education	1.27×10^−08^ (−3.59×10^−08^–6.12×10^−08^)	−2.30×10^−08^ (−1.03×10^−07^–5.66×10^−08^)	−1.06×10^−07^ (−2.49×10^−07^–3.65×10^−08^)
Energy	−2.33×10^−09^ (−3.48×10^−08^–3.01×10^−08^)	−3.20×10^−08^ (−8.62×10^−08^–2.22×10^−08^)	−1.03×10^−07^** (−2.04×10^−07^−1.80×10^−09^)
Transportation	−1.86×10^−09^ (−4.19×10^−08^–3.82×10^−08^)	−2.95×10^−08^ (−9.49×10^−08^–3.58×10^−08^)	−9.74×10^−08^ (−2.19×10^−07^–2.46×10^−08^)
Durable items	3.27×10^−08^*** (1.04×10^−08^–5.51×10^−08^)	3.07×10^−08^* (−5.82×10^−09^–6.72×10^−08^)	1.94×10^−08^ (−4.50×10^−08–^8.37×10^−08^)
Personal services and entertainment	2.78×10^−09^ (−3.49×10^−08^–4.05×10^−08^)	−3.52×10^−08^ (−9.88×10^−08^–2.84×10^−08^)	−1.26×10^−07^** (−2.49×10^−07–^-3.89×10^−09^)
Other OOP health expenditure^b^	−	4.79×10^−07^*** (3.33×10^−07^–6.25×10^−07^)	1.85×10^−06^*** (1.35×10^−06^–2.36×10^−06^)
	***n*=4213**	***n*=4213**	***n*=4213**

NCD: non-communicable disease; OOP: out-of-pocket.

Estimates are weighted and based on the Nigeria Living Standard Survey 2018/2019.

Estimates are adjusted for household socioeconomic and demographic characteristics.

95% CIs are shown in parentheses.

Standard errors are robust to heteroscedasticity.

***p<0.01; **p<0.05; *p<0.1.

a Staples represent rice, maize, grain, baked and starchy foods; protein represents meat, fish, poultry, dairy and pulses; beverages represent alcoholic and non-alcoholic drinks; and entertainment includes newspapers, magazines, cinemas, leisure and tobacco.

b Other OOP health expenditure represents the remaining OOP health expenditure after deduction of non-NCD OOP expenditure (i.e. NCD OOP health expenditure) or after deduction of NCD OOP expenditure (i.e. non-NCD OOP health expenditure) as the case may be.

Non-NCD OOP expenditure crowded out clothing/footwear, fruits/vegetables and oil/other food categories. NCD OOP expenditure crowded out clothing/footwear, personal services/entertainment and all essential items (all food categories, energy). However, housing and education were protected. Discretionary items (transportation and durables) were also protected.

NCD and non-NCD OOP expenditures crowded each other in, with a greater magnitude for NCD OOP expenditure (1.85×10^−06^ [95% CI 1.35×10^−06^ to 2.36×10^−06^] vs 4.79×10^−07^ [95% CI 3.33×10^−07^ to 6.25×10^−07^]).

The magnitude of crowding-out effects varied. For example, a US$1 increase in OOP health expenditure crowded out clothing/footwear by 2.93×10^−08^ (95% CI −5.17×10^−08^ to −6.80×10^−09^) pp, or −2.93×10^−08^ × M US$, where M is the remaining budget after OOP health expenditure (Table 1). NCD OOP expenditure had a greater crowding-out effect than non-NCD or total OOP expenditure.

### Adjusted crowding-out effect of non-communicable disease out-of-pocket health expenditure by socioeconomic status

Table 3 shows the crowding-out effect of NCD OOP expenditure by SES. Protein and oil/other food categories were crowded out in lower and higher SES households, while staples, meals outside the home and education were crowded out in the middle SES. Fruits/vegetables and energy were crowded out in lower SES, while sugar/beverages were crowded out in higher SES. Clothing/footwear was crowded out in both lower and higher SES. Housing, education, transportation and durable items were protected across all SES. Staples, sugar/beverages, meals outside home and education were protected in the lowest SES. Middle SES protected sugar/beverages, fruits/vegetables, energy, protein, clothing and footwear. Higher SES protected staples. The degree of crowding out was greatest in the lowest SES. For instance, crowding out of protein and oil/other food was 3.4 and 8 times greater, respectively, in the lowest compared with the highest SES.

**Table 3.** Adjusted crowding-out effect of NCD OOP health expenditure by socioeconomic status

	Socioeconomic status^a^		
	Lower (95% CI)	Middle (95% CI)	Upper (95% CI)
Item name	Coefficient	Coefficient	Coefficient
Food (at home)^b^			
Staples	−7.05×10^−07^ (−1.64×10^−06^–2.26×10^−07^)	−5.57×10^−07^*** (−9.06×10^−07–^-2.08×10^−07^)	1.30×10^−08^ (−1.32×10^−07^–1.57×10^−07^)
Protein	−7.08×10^−07^** (−1.35×10^−06^−6.74×10^−08^)	−1.67×10^−07^ (−5.18×10^−07^–1.84×10^−07^)	−2.06×10^−07^** (−4.10×10^−07^−1.58×10^−09^)
Fruits and vegetables	−8.18×10^−07^*** (−1.30×10^−06^−3.32×10^−07^)	−1.01×10^−07^ (−2.62×10^−07^–5.91×10^−08^)	−6.50×10^−08^* (−1.32×10^−07^–2.04×10^−09^)
Sugar and beverages	−1.03×10^−07^ (−3.28×10^−07^–1.21×10^−07^)	−4.01×10^−08^ (−1.24×10^−07^–4.37×10^−08^)	−1.01×10^−07^*** (−1.69×10^−07^−3.24×10^−08^)
Oil and others	−4.69×10^−07^*** (−7.80×10^−07^−1.58×10^−07^)	−7.59×10^−08^ (−1.76×10^−07^–2.39×10^−08^)	−5.83×10^−08^** (−1.15×10^−07^−1.96×10^−09^)
Meals (outside home)	−8.30×10^−07^* (−1.68×10^−06^–2.01×10^−08^)	−3.71×10^−07^** (−7.11×10^−07^−3.05×10^−08^)	−2.39×10^−08^ (−2.07×10^−07^–1.59×10^−07^)
Housing	−5.58×10^−08^ (−2.08×10^−07^–9.65×10^−08^)	2.82×10^−08^ (−5.89×10^−08^–1.15×10^−07^)	9.37×10^−09^ (−4.73×10^−08^–6.61×10^−08^)
Clothing and footwear	−4.10×10^−07^*** (−6.95×10^−07^−1.26×10^−07^)	−6.87×10^−08^ (−1.70×10^−07^–3.23×10^−08^)	−1.21×10^−07^*** (−2.05×10^−07^−3.70×10^−08^)
Education	−1.43×10^−08^ (−4.02×10^−07^–3.74×10^−07^)	−3.37×10^−07^*** (−5.75×10^−07^−9.90×10^−08^)	−3.09×10^−08^ (−1.89×10^−07^–1.27×10^−07^)
Energy	−4.12×10^−07^** (−7.47×10^−07^−7.63×10^−08^)	−7.32×10^−08^ (−2.28×10^−07^–8.16×10^−08^)	−9.18×10^−09^ (−1.16×10^−07^–9.81×10^−08^)
Transportation	−1.34×10^−08^ (−4.22×10^−07^–3.96×10^−07^)	−1.12×10^−07^ (−2.95×10^−07^–7.18×10^−08^)	−1.18×10^−07^ (−2.60×10^−07^–2.34×10^−08^)
Durable items	−1.24×10^−08^ (−2.31×10^−07^–2.06×10^−07^)	2.29×10^−08^ (−6.19×10^−08^–1.08×10^−07^)	3.34×10^−08^ (−4.09×10^−08^–1.08×10^−07^)
Personal services and entertainment	−1.92×10^−07^ (−4.40×10^−07^–5.61×10^−08^)	−5.67×10^−08^ (−1.86×10^−07^–7.28×10^−08^)	−7.41×10^−08^ (−2.27×10^−07^–7.87×10^−08^)
OOP health expenditure on non-NCDs	4.58×10^−06^*** (3.07×10^−06^–6.09×10^−06^)	1.83×10^−06^*** (1.29×10^−06^–2.36×10^−06^)	7.44×10^−07^*** (3.50×10^−07^–1.14×10^−07^)
	***n*=1961**	***n*=1476**	***n*=774**

NCD: non-communicable disease; OOP: out-of-pocket.

Estimates are weighted and based on the Nigeria Living Standard Survey 2018/2019.

Estimates are adjusted for household socioeconomic and demographic characteristics.

95% CIs are shown in parentheses.

Standard errors are robust to heteroscedasticity.

***p<0.01; **p<0.05; *p<0.1.

a Tertiles of adjusted household expenditure per capita.

b Staples represent rice, maize, grain, baked and starchy foods; protein represents meat, fish, poultry, dairy and pulses; beverages represent alcoholic and non-alcoholic drinks; and entertainment includes newspapers, magazines, cinemas, leisure and tobacco.

Non-NCD OOP expenditure was protected across all SES, with the largest effect being in the lowest SES.

## Discussion

This study investigates the crowding-out effect of NCD OOP health expenditure on household consumption in Nigeria. NCD OOP expenditure constitutes a substantial portion of household budgets, particularly impacting lower SES households. While higher SES households allocate a smaller budget share to food, all SES groups experience a crowding-out effect on essential consumption items (excluding non-NCD OOP health expenditure), with lower SES households disproportionately affected.

Our findings are consistent with studies demonstrating the association between high OOP health expenditures and reduced expenditure shares for food and non-food consumption.^5,6,10,11^ Despite representing a small fraction (<2%) of household budgets, NCD OOP expenditure disproportionately negatively impacted other consumption categories, particularly among lower socioeconomic groups. This aligns with research showing a greater crowding-out effect of NCD-related spending on essential items like food and education.^7,12^ In resource-poor contexts with already tight budgets, even a relatively small OOP health spending can disproportionately reduce spending on food, education and other essential consumption, exacerbating food insecurity and perpetuating poverty cycles.^5,10,12,28^ This underlines the importance of reducing OOP burdens and strengthening social protection.

While OOP protected essential consumption, NCD spending crowded out all food categories, and non-NCD spending conversely crowded out fruits and vegetables, a finding with significant implications for the NCD burden in Nigeria, given the established protective role of these food groups.^29^ This pervasive compromise in dietary quantity and quality due to NCD expenditures echoes findings from Bangladesh.^7^ This portends profound implications for household food security, health and economic well-being. The crowding out of protein, fruits and vegetables creates a double burden: it not only hinders NCD prevention and management, but also risks perpetuating a cycle of ill-health and escalating healthcare costs and poverty.^30,31^ Protein deficiency, crucial for muscle mass, immune function and NCD prevention and treatment,^32^ can have long-term consequences for growth, development and infection susceptibility, particularly in children, exacerbating the double burden of infectious diseases and NCDs. Furthermore, the crowding out of staples threatens essential nutrition. Overall, household dietary compromise due to NCD spending can escalate the NCD burden and diminish welfare through reduced productivity and adverse health outcomes.^33^

We observed disparities in the crowding-out effect of NCD OOP expenditure across SES groups. While all groups experience this effect, lower SES households are most severely impacted, mainly through compromised access to healthy foods and energy sources. The magnitude of the crowding-out effect is also greater for these households. This also aligns with previous studies.^7,12^ Food, especially staples and fruits/vegetables, constitutes a larger share of total household expenditure for lower and middle SES households, despite lower absolute food spending than higher SES households. This is consistent with the established pattern of substantial food budget shares among poorer households in low- and middle-income countries.^34^ Yet, even these essential food expenditures are compromised by NCD spending.

The crowding out of energy (impacting cooking fuel access) has critical implications for NCD management and broader public health policy. Limited access to clean cooking energy is linked to food insecurity in Nigeria.^35^ This may explain the observed protection of spending on less healthy options (alcohol, sugary drinks and meals consumed outside the home) among lower SES households. Reliance on these readily available but unhealthy and often expensive alternatives can exacerbate nutritional deficiencies and chronic health problems, undermining long-term management of NCDs.^33,36^

Middle SES households were also significantly affected, experiencing crowding out of staples, as was observed in Bangladesh,^7^ and education expenditures. The impact on staples threatens basic nutrition essential for energy and productivity, with repercussions for household welfare.^37^ The displacement of education spending suggests a struggle among middle-income earners to maintain access to expensive private education despite resource constraints, mirroring findings from Pakistan.^12^ These impacts on nutrition and education can have long-term consequences for children, perpetuating economic disparities across generations.

Rent and transportation protection likely reflects necessity, particularly for individuals managing NCDs. Stable housing facilitates recovery, while transportation enables access to healthcare, enabling access to services, medication refills and follow-up appointments, which are essential for effective NCD management.^38–40^ Durables that are less frequently purchased may have been prioritised over recurring OOP health expenses. Interestingly, discretionary spending on sugar, alcoholic and sugary beverages, entertainment (including tobacco) and meals outside the home remained relatively unaffected by OOP expenditures for NCDs in lower SES households. This contradicts studies suggesting that health spending crowds out these expenditures.^20^ One plausible explanation, although not directly tested, is that these items may serve as coping mechanisms for these households, potentially substituting for healthier or home-cooked options when access to cooking fuel is limited. There is an established association between smoking, sugar-sweetened beverages, alcohol and ready-made food, and a lower consumption of healthy food.^18,41,42^

Additionally, inadequate cooking habits have been linked to the consumption of an unhealthy diet.^43,44^ Another possible explanation may be limited awareness of the health consequences of these choices. Nevertheless, future qualitative research could explore the underlying reasons behind these spending patterns to inform interventions to promote healthier behaviours and reduce the financial burden of NCDs.

The observed bidirectional crowding-out effects between NCD and non-NCD OOP expenditure, similar to previous studies,^12^ may suggest multimorbidity, complementary health-seeking behaviour or increased healthcare utilisation.

### Strengths of the study

This study’s strengths include using a large, nationally representative dataset capturing detailed household consumption and health expenditure information. The application of rigorous econometric techniques, using the GMM and the QUAIDS, enables unbiased estimates of crowding-out effects while accounting for endogeneity. Furthermore, by disaggregating NCD and non-NCD expenditures and stratifying households by socioeconomic status, this study advances our understanding of the differentiated impacts of NCD-related expenditure on vulnerable populations. These methodological choices enhance the robustness and policy relevance of our findings.

### Limitations of this study

Despite these strengths, our study’s limitations include the lack of longitudinal data to explore long-term crowding-out effects, potential recall bias from self-reported data and the absence of an analysis of health insurance protective impacts, given Nigeria’s low coverage.^3^ The data used in this study are not very recent; however, they offer valuable insights into emerging trends and establish a baseline for future research on the crowding-out effect of NCD OOP expenditure, using more current data. Although it is beyond the scope of this study, further qualitative research is needed to provide a more in-depth understanding and nuanced interpretation of our findings. While this study provides a learning opportunity for SSA countries with similarly high OOP health expenditures and a growing burden of NCDs, such as Nigeria, caution is required when generalising the findings to all SSA countries.

## Conclusions

This study highlights the critical need for increased public health funding and universal health targeting NCDs and vulnerable households in Nigeria. Innovative financing mechanisms, such as taxes on unhealthy commodities (tobacco, alcohol, sugar), should be considered. We emphasise the potential long-term consequences of the crowding-out effect of NCDs on household welfare. Our research suggests that reducing OOP expenditure alone is insufficient; promoting healthier behaviours through health education, addressing food insecurity and ensuring access to affordable, clean cooking fuel are also essential. Future research should shift from cross-sectional quantitative analyses to longitudinal and mixed-methods approaches. Longitudinal data would clarify the causal pathways and temporal dynamics of the effect of OOP health expenditure displacement on other consumption. Qualitative methods can provide insights into household coping strategies and decision-making processes related to constrained consumption. This integration is essential for developing and evaluating effective health financing and social protection policies to address NCD-related impoverishment in Nigeria and SSA.

## Supplementary Material

ihaf117_Supplemental_File

## Data Availability

The data supporting this study’s findings are available in the World Bank microdata library at https://microdata.worldbank.org/index.php/catalog/3827/get-microdata.
