# Supplementary material for: Crowding-out effect of out-of-pocket expenditure on non-communicable diseases in sub-Saharan Africa: a Nigerian case study
Source: Int Health. 2025 Nov 20;18(4):629–40. doi: 10.1093/inthealth/ihaf117 (PMC13329968; doi:10.1093/inthealth/ihaf117)
Supplement: ihaf117_Supplemental_File [file ihaf117_supplemental_file.docx]

***Supplementary Table 1*: Statistical Test (Total Out-of-pocket Health Expenditure)**

| Household Consumption Items^β^ | | | | | | | | | | | | |  |
| --- | --- | --- | --- | --- | --- | --- | --- | --- | --- | --- | --- | --- | --- |
| Test^α^ | Staples | Protein | Fruits & Vegetables | Sugar & Beverages | Oil & Others | Meals | Housing | Clothing & footwear | Education | Energy | Transportation | Durable Items | Personal Services & Entertainment |
| Household  preference test  (Wald tests,  p-value) | 1.45  (0.6946) | 1.10  (0.7764) | 1.03  (0.7948) | 1.04  (07919) | 2.33  (05064) | 1.76  (0.6245) | 1.29  (0.7306) | 2.78  (0.4096) | 2.89  (0.4096) | 1.68  (06414) | 1.21  (0.7507) | 2.38  (0.4971) | 1.31  (0.7265) |
| Heteroskedasticity  test  (Pagan-Hall general  test statistic, p-value) | 234.377 (0.0000) | 109.374 (0.0000) | 76.752 (0.0000) | 98.454 (0.0000) | 119.565  (0.0000) | 95.019  (0.0000) | 82.308  (0.0000) | 133.892  (0.0000) | 384.034  (0.0000) | 52.533  (0.0048) | 46.357  (0.0216) | 54.765  (0.0026) | 228.403 (0.0000) |
| Breusch–Pagan Lagrange Multiplier tests (Overall) | 3179.167  (0.0000) | 3179.167  (0.0000) | 3179.167  (0.0000) | 3179.167  (0.0000) | 3179.167  (0.0000) | 3179.167  (0.0000) | 3179.167  (0.0000) | 3179.167  (0.0000) | 3179.167  (0.0000) | 3179.167  (0.0000) | 3179.167  (0.0000) | 3179.167  (0.0000) | 3179.167  (0.0000) |
| Cragg-Donald Wald F-statistic for first-stage regression test | 41.925  (0.0000) | 41.925  (0.0000) | 41.925  (0.0000) | 41.925  (0.0000) | 41.925  (0.0000) | 41.925  (0.0000) | 41.925  (0.0000) | 41.925  (0.0000) | 41.925  (0.0000) | 41.925  (0.0000) | 41.925  (0.0000) | 41.925  (0.0000) | 41.925  (0.0000) |
| Under  identification test  (Kleibergen-Paap rank LM-test, p-value) | 68.285  (0.0000) | 68.285  (0.0000) | 68.285  (0.0000) | 68.285  (0.0000) | 68.285  (0.0000) | 68.285  (0.0000) | 68.285  (0.0000) | 68.285  (0.0000) | 68.285  (0.0000) | 68.285  (0.0000) | 68.285  (0.0000) | 68.285  (0.0000) | 68.285  (0.0000) |
| Weak  identification test  (Kleibergen-Paap rank Wald F statistic) | 23.435  (0.0000) | 23.435  (0.0000) | 23.435  (0.0000) | 23.435  (0.0000) | 23.435  (0.0000) | 23.435  (0.0000) | 23.435  (0.0000) | 23.435  (0.0000) | 23.435  (0.0000) | 23.435  (0.0000) | 23.435  (0.0000) | 23.435  (0.0000) | 23.435  (0.0000) |
| Endogeneity test  (GMM-C-Statistics,  p-value) | 0.492  (0.9206) | 2.285  (0.5154) | 3.574  (0.3113) | 2.323  (0.5081) | 4.036  (0.2577) | 17.439  (0.0006) | 2.713  (0.4381) | 8.253  (0.0411) | 2.149  (0.5420) | 3.881  (0.2747) | 1.622  (0.6544) | 15.652  ( 0.0013) | 3.915  (0.2708) |
| Notes: Estimates are weighted and based on Nigeria Living Standard Survey 2018/2019.  ^α^ All tests were performed with non-health expenditure budget share of each consumption item as the dependent variable.  ^β^ *staple* represents rice, maize, grain, baked & starchy foods; protein represents meat, fish, poultry, dairy and pulse; and *beverages* represent alcoholic & non-alcoholic drinks.  NCDs mean non-communicable diseases and OOPHE means out-of-pocket health expenditure  P- values are shown in parentheses.  Standard errors are robust to heteroscedacity. | | | | | | | | | | | | | |

**Supplementary Table 2: Unadjusted difference between out-of-pocket health spender and non-spender households for all diseases, non-NCDs and NCDs in Nigeria, 2018/19**

| **Difference in Means between Non-spenders and Spenders** | | |  |
| --- | --- | --- | --- |
| **Items Name ^α^** | **Total OOP health expenditure**  **(95%CI)** | **Non-NCD OOP health expenditure**  **(95%CI)** | **NCD OOP health expenditure**  **(95%CI)** |
| Food (at home) |  |  |  |
| Staples | 4.51×10^-03^*  (-2.82×10^-04^-9.31×10^-03^) | 1.23×10^-03^  (-2.83×10^-03^-5.28×10^-03^) | 5.10×10^-04^  (-3.98×10^-03^-5.00×10^-03^) |
| Protein | -6.48×10^-03^***  (-9.82×10^-03^--3.14×10^-03^) | -1.95×10^-03^  (-4.73×10^-03^-8.31×10^-04^) | -6.70×10^-03^***  (-1.00×10^-02^--3.39×10^-03^) |
| Fruits and Vegetables | -3.02×10^-03^***  (-4.90×10^-03^--1.15×10^-03^) | -2.53×10^-03^***  (-4.08×10^-03^--9.78×10^-04^) | -2.59×10^-03^***  (-4.30×10^-03^--8.71×10^-04^) |
| Sugar and beverages | -2.57×10^-03^***  (-3.61×10^-03^--1.53×10^-03^) | -1.56×10^-03^***  (-2.43×10^-03^--6.88×10^-04^) | -1.96×10^-03^***  (-2.91×10^-03^--9.99×10^-04^) |
| Oil and others | -7.55×10^-04^  (-2.12×10^-03^-6.13×10^-04^) | -1.56×10^-03^***  (-2.72×10^-03^--4.03×10^-04^) | -3.43×10^-03^***  (-4.67×10^-03^--2.20×10^-03^) |
| Meals (outside home) | -2.61×10^-02^***  (-3.20×10^-02^--2.02×10^-02^) | -2.09×10^-02^***  (-2.56×10^-02^--1.61×10^-02^) | -1.86×10^-02^***  (-2.36×10^-02^--1.36×10^-02^) |
| Housing | -1.00×10^-02^***  (-1.21×10^-02^--7.95×10^-03^) | -7.54×10^-03^***  (-9.20×10^-03^--5.89×10^-03^) | -7.71×10^-03^***  (-9.36×10^-03^--6.05×10^-03^) |
| Clothing and footwear | -7.21×10^-03^***  (-8.55×10^-03^--5.88×10^-03^) | -5.04×10^-03^***  (-6.15×10^-03^--3.93×10^-03)^ | -8.08×10^-03^***  (-9.24×10^-03^--6.91×10^-03^) |
| Education | 4.20×10^-03^***  (1.34×10^-03^-7.06×10^-03^) | 4.51×10^-03^***  (2.12×10^-03^-6.90×10^-03^) | 3.69×10^-04^  (-2.43×10^-03^-3.17×10^-03^) |
| Energy | -9.37×10^-03^***  (-1.12×10^-02^--7.52×10^-03^) | -6.79×10^-03^***  (-8.37×10^-03^--5.20×10^-03^) | -8.22×10^-03^***  (-1.01×10^-02^--6.34×10^-03^) |
| Transportation | -1.40×10^-02^***  (-1.70×10^-02^-1.10×10^-02^) | -9.33×10^-03^***  (-1.18×10^-02^--6.85×10^-03^) | -1.02×10^-02^***  (-1.26×10^-02^--7.76×10^-03^) |
| Durable items | -2.04×10^-03^***  (-3.10×10^-03^--9.75×10^-04^) | -1.71×10^-03^***  (-2.62×10^-03^--8.01×10^-04^) | -5.61×10^-04^  (-1.69×10^-03^-5.65×10^-04^) |
| Personal services and entertainment | -1.05×10^-02^***  (-1.25×10^-02^--8.41×10^-03^) | -7.52×10^-03^***  (-9.30×10^-03^--5.74×10^-03^) | -9.22×10^-03^***  (-1.13×10^-02^--7.13×10^-03^) |
|  | ***n*=22,110** | ***n*=22,110** | ***n*=22,110** |
| *Notes:* Estimates are weighted and based on the Nigeria Living Standard Survey 2018/2019.  Estimates are adjusted for household socioeconomic and demographic characteristics.  ***^α^*** *Staple* represents rice, maise, grain, baked & starchy foods; protein represents meat, fish, poultry, dairy and pulse; *beverages* represents alcoholic & non-alcoholic drinks; and *entertainment* includes newspapers, magazines, cinemas, leisure and tobacco.  *NCDs* means non-communicable diseases, and *OOP health expenditure* means out-of-pocket health expenditure.  95% CIs are shown in parentheses.  *P* *values:* *** p<0.01, ** p<0.05, * p<0.1 | | | |
